# Fluid intake of Latin American children and adolescents: results of four 2016 *LIQ.IN*^*7*^ National Cross-Sectional Surveys

**DOI:** 10.1007/s00394-018-1728-8

**Published:** 2018-06-01

**Authors:** J. Gandy, H. Martinez, E. Carmuega, J. L. Arredondo, C. Pimentel, L. A. Moreno, S. A. Kavouras, J. Salas-Salvadó

**Affiliations:** 1 British Dietetic Association, Birmingham, UK; 2 School of Life and Medical Services, University of Hertfordshire, Hatfield, AL10 9AB UK; 3 Hospital Infantil de México Federico Gómez, Mexico City, Mexico; 4 Center of Studies on Infant Nutrition, Buenos Aires, Argentina; 5 Unidad de Apoyo a la Investigación Clínica, Instituto Nacional de Pediatría, Mexico City, Mexico; 6 GENUD (Growth, Exercise, NUtrition and Development) Research Group, Faculty of Health Sciences, Universidad de Zaragoza, Instituto Agroalimentario de Aragón (IA2), Instituto de Investigación Sanitaria Aragón (IIS Aragón), Zaragoza, Spain; 7 CIBERobn (Centro de Investigación Biomédica en Red Fisiopatología de la Obesidad y Nutrición), Institute of Health Carlos III, Madrid, Spain; 8 Hydration Science Lab, University of Arkansas, Fayetteville, AR USA; 9 Division of Endocrinology, University of Arkansas for Medical Sciences, Little Rock, AR USA; 10 Human Nutrition Unit, Hospital Universitari de Sant Joan de Reus, Faculty of Medicine and Health Sciences, Institut d’Investigació Sanitària Pere Virgili, Biochemistry and Biotechnology Department, Universitat Rovira i Virgili, C/Sant Llorenç, 21, 43201 Reus, Spain

**Keywords:** Beverages, Fluid intake, Water, Hydration, *Liq.in*^*7*^, Children, Adolescents, Mexico, Brazil, Uruguay, Argentina

## Abstract

**Purpose:**

The primary aim of this survey was to report total fluid intake (TFI) and different fluid types for children (4–9 years) and adolescents (10–17 years) in Mexico, Brazil, Argentina and Uruguay. The second aim was to compare TFI with the adequate intake (AI) of water from fluids as recommended by the USA Institute of Medicine.

**Methods:**

Data were collected using a validated liquid intake 7-day record (*Liq.In*^*7*^). Participants’ characteristics, including age, sex and anthropometric measurements were recorded.

**Results:**

A total of 733 children and 933 adolescents were recruited. Over 75% of children in Uruguay met the IOM’s recommended intake. Fewer children in Argentina (64–72%) and Brazil (41–50%) obtained AI and the lowest values were recorded in Mexico (33–44%), where 16% of boys and 14% girls drank 50% or less of the AI. More adolescents in Argentina (42%) met the AIs than other countries; the lowest was in Mexico (28%). Children and adolescents in Mexico and Argentina drank more sugar sweetened beverages than water.

**Conclusions:**

Large numbers of children and adolescents did not meet AI recommendations for TFI, raising concerns about their hydration status and potential effects on mental and physical well-being. Given the negative effects on children’s health, the levels of SSB consumption are worrying.

**Electronic supplementary material:**

The online version of this article (10.1007/s00394-018-1728-8) contains supplementary material, which is available to authorized users.

## Introduction

Inadequate hydration in children and adolescents has been shown to affect both physical [1] and cognitive performance [2, 3]. However, few studies have looked at children or adolescents’ fluid intake in terms of total volume and adequacy in Latin America. Piernas et al. [4] reported total water intakes (TWI) (sum of food moisture and fluid intake) in children and adolescents in the 2012 Mexican National Health and Nutrition survey. Alarmingly a high proportion of the subjects, 71% of 4–6-year-old, 81–83% of 9–13-year-old and 83–87% of 14–18-year-old did not meet the USA Institute of Medicine (IOM) recommendations [5] for the adequate intake (AI) of total water. Information was not collected on hydration status but given the high number of participants not meeting the recommendations it is likely that some, if not most, were at risk of the effects of hypohydration. This was emphasized by data collected from a fluid intake survey (*Liq.In*^*7*^) in Mexico that reported 54–65% of 4–9-year-old and 55 to greater than 70% of 10–17-year-old as having fluid intakes less than the recommended adequate intakes [6]. While these levels are lower than the Piernas et al. study [4], they reinforce concerns about the potential risk of the effects of low fluid intake on the health and well-being of this population. This *Liq.In*^*7*^ study also reported the percentages of children (4–9 years) and adolescents (10–17 years) in Brazil, Uruguay and Argentina not reaching the recommended intakes of fluids as 32, < 20 and < 37%, respectively, for children and 35–50, 15–23 and 45–70% for adolescents, respectively. While these levels are lower than those observed in Mexico, there is still potential cause for concern especially in Argentina.

Several other studies have been conducted in Latin America assessing fluid intake of children and adolescents. For example, total fluid intake and fluid types have been reported in Mexico [7], and Brazil [8]; however, the emphasis has been on the energy content of fluids rather than adequate intakes. Other studies have only reported energy-containing fluids and have not included non-caloric fluids including water [9]. This emphasis on energy from fluids is the result of concerns about the increasing levels of overweight and obesity in this region [10–12] and the need to develop effective interventions. A recent review highlighted this and other negative effects of some beverages on children [13]. Surveys of fluid intake, in terms of both volume and type of fluid consumed, are a vital part of the process of developing public health policies and interventions aimed at improving the health of these vulnerable populations. This is particularly important in Latin America where there is a paucity of such data.

It is important to recognize that the choice of recommendations used for comparisons between survey results will influence the findings and conclusions. The study of Piernas et al. [4] used the recommendations for the USA IOM for total water. The *Liq.In*^*7*^ [6] was an intercontinental study; therefore, the EFSA recommendations [14] were used as they are more conservative than those of the IOM [5] and less likely to overestimate non-adherence. There is no agreed methodology for the development of recommendations on the adequate intake of water and different approaches to establishing such recommendations have been taken [15]. For example, the IOM recommendations are based on median intakes from national surveys while the EFSA recommendations are based on population studies and other factors including desirable osmolarity values of urine and desirable water volumes per unit energy consumed. Specific recommendations are not available for Latin American countries; therefore, the choice of recommendations for comparison is subjective. The present study resurveyed samples of the child and adolescent populations from Argentina, Brazil, Mexico and Uruguay. Unlike the former survey [6], it was focused entirely on Latin American countries; therefore, the IOM recommendations were used to assess adherence to AI of fluids in these populations in line with other studies as discussed above.

Therefore, the primary objective of the present study was to report total fluid intake (TFI) and intake of different fluid types of children (4–9 years) and adolescents (10–17 years) in Mexico, Brazil, Argentina and Uruguay. The secondary aim was to compare TFI with the AI recommendations set by the USA IOM [5].

## Methods

### Design and study population

The present analysis reports cross-sectional surveys of children aged 4–9 years (6–9 years old in Uruguay) and adolescents (10–17 years) in Argentina, Brazil, Mexico and Uruguay. The age ranges were chosen as it was felt, after consultation with pediatricians, that children less than 10 years of age could be considered prepubescent. One parent, or care giver, recorded data for children < 12 years old, while older adolescents self-reported the amount and types of beverage consumed. These surveys are part of a multinational project called *Liq.In*^*7*^ The primary objective of the *Liq.In*^*7*^ surveys is to assess the sources of fluid consumption, including drinking water and different types of beverages. To ensure harmony across the surveys standard operating procedures related to the method of recruitment, the instruments for data collection and data treatment were developed by the coauthors and a central research private organization, and then distributed to local investigators of this private research organization. The data collection was performed in 2016 between March and May in different regions of Argentina, Mexico, and Uruguay; and for operational reasons between November and December in Brazil.

Participants were recruited via a systematic door-to-door recruitment until suitable quotas for age, sex, region and socioeconomic characteristics, in relation to the total country population, were met. Only one individual per household was eligible to participate. If several individuals of one household were eligible, the investigator selected the individual based on whether or not the quotas had already been achieved. Inclusion criteria were apparently healthy individuals. Participants who had a parent or caregiver who was illiterate, or those working in any capacity in a company in anyway associated with the manufacture, distribution and/or sale of water and any other kind of beverage were excluded from participation. Pregnancy and lactation were not exclusion criteria. After receiving a detailed description of the study and its objectives, following the principles of informed consent, participants’ parent or guardian gave oral approval of their willingness to be included. No monetary incentive was offered for taking part in the study. All data were recorded anonymously.

### Ethical approval

The survey protocol was reviewed and approved by the University of Arkansas Review Board (ref. 14-12-376).

### Anthropometry

Height (m) and weight (kg) were self-reported by participants or care givers depending on the participant’s age. The body mass index (BMI) *z* score was calculated (kg/m^2^).

### Assessment of total fluid intake and the different fluid types

Participants aged 12–17 years of age or the parent or caregiver of children aged 4–11 years completed the *Liq.In*^*7*^ record in the official language of the country. The *Liq.In*^*7*^ record is a 7-day fluid-specific record validated for accuracy and reliability [16]. The *Liq.In*^*7*^ record consists of a grid structured according to different times of the day from waking, meal times (breakfast, lunch and dinner) and periods between meals (morning, before lunch/aperitif, afternoon, tea break, before dinner/aperitif, evening, just before going to bed) to during the night. The participants were instructed to record on this grid all drinking events at any moment of the day with the following details; the fluid type, the volume consumed, the size of the container from which it was drunk, where it was drunk and whether food was also consumed. Food consumption was not reported. The record was accompanied by a booklet with pictures of standard fluid containers to assist the estimation of the amount of fluid consumed.

Before the survey began, the researcher explained use of the record in an initial face-to-face interview in the participant’s home. After a period of 7 days, the record was collected by the researcher and checked for completion with the participant and/or parent/caregiver. Participants who did not complete the full 7 days of the fluid record, who reported a mean total daily fluid intake below 0.4 L/day or higher than 6 L/day for children aged 14–17 years and higher than 4 L/day for children under the age of 14 years, were excluded from the analysis.

### Classification and analysis of the fluid types

Fluids recorded were classified as water (tap and bottled water), milk and milk derivatives, hot beverages (coffee, tea and other), 100% fruit juices, sugar sweetened beverages (SSB) (carbonated soft drinks (CSDs), juice-based drinks, functional beverages such as energy and sports drinks, ready to drink tea and coffee and flavored water), artificial/non-nutritive sweeteners beverages (A/NSB) (diet/zero/light soft drinks), alcoholic drinks and other beverages. The water and milk content of hot beverages, including “mate”, were not disaggregated. More details of the fluid categories can be found in supplementary Table S2. TFI was defined as the sum of all these categories. In Uruguay and Argentina only, a specific code for the fluid type “mate” was included as previous surveys had indicated a significant daily intake of this traditional drink and this was considered of interest by local collaborators. A participant was defined as a consumer of a certain fluid type if this fluid type was consumed at least once during the 7-day period.

Individual’s estimated daily TFI was compared with the AI for water from fluids (beverages including drinking water) set by the USA IOM [5]. To allow comparison with previously published data, the comparison between observed intakes and the recommendations set by  EFSA [14] is provided in the supplementary materials (Figure S1). The numbers of individuals drinking ≤ 1 serving (being 250 mL) of SSB per week, 2–6 servings of SSB per week and ≥ 1 serving/day intake of SSB was recorded. These cut-offs were obtained from meta-analyses associating such levels of intake with potential risks for the development of obesity, type 2 diabetes and metabolic syndrome [17–19].

### Statistical analysis

The demographic and anthropometric characteristics of the study population are presented either as means and standard deviations (SD) for continuous variables, or numbers and percentages for dichotomous variables. TFI are presented as median (25th–75th percentiles) and mean [standard error of mean (SEM)]. Due to the skew in intakes (supplementary figure S2), the different fluid types are presented as median (50th percentile), 25th–75th percentiles and proportion of consumers. The presented contribution (%) to TFI was calculated from the mean intake of each fluid types. The median (25th–75th percentiles) and mean (SEM) of the different fluid types by sex and age group can be found as supplementary table S2 and S3, respectively. Between sex comparisons were made with a Wilcoxon rank test for continuous variables. All statistical tests were two-tailed and the significance level was set at *P* < *0.05*. All analyses were performed using the SPSS software version 22.0 (SPSS Inc, Chicago, IL) and were verified by a statistician.

## Results

### Participant characteristics

The characteristics of the populations surveyed are shown in Table 1. A total of 733 children (4–9 years) and 933 adolescents (10–17 years) were recruited. The sexes were represented evenly overall although there were some disparities within the countries and age groups.

**Table 1:** Demographic and anthropometric characteristics of the study population, by country and age group

	4–9 years			6–9 years	10–17 years			
	Mexico	Brazil	Argentina	Uruguay	Mexico	Brazil	Argentina	Uruguay
Sample size^a^	293	146	173	121	376	194	219	144
Male	140 (48)	68 (47)	114 (66)	57 (47)	212 (56)	62 (32)	130 (59)	74 (51)
Female	153 (52)	78 (53)	59 (34)	64 (53)	164 (44)	132 (68)	89 (41)	70 (49)
Age^b^ (years)	6.5 ± 1.7	6.6 ± 1.8	6.4 ± 1.7	7.7 ± 0.9	13.5 ± 2.4	13.6 ± 2.3	13.5 ± 2.2	12.3 ± 2.3
Weight^b^ (kg)	31.5 ± 13.3	27.4 ± 8.5	28.1 ± 10.6	35.0 ± 10.1	51.8 ± 13.2	53.3 ± 16.4	52.9 ± 13.6	46.7 ± 15.2
Height^b^ (m)	1.2 ± 0.2	1.2 ± 0.1	1.2 ± 0.2	1.4 ± 0.1	1.5 ± 0.2	1.6 ± 0.1	1.6 ± 0.1	1.5 ± 0.1
BMI *z* score^b^	2.6 ± 3.2	1.2 ± 1.8	1.4 ± 2.1	1.1 ± 1.8	0.9 ± 1.3	0.5 ± 1.4	0.6 ± 1.2	0.5 ± 1.3

*BMI* body mass index

^a^Data presented as numbers (percentage) for dichotomous variables

^b^Data presented as mean ± standard deviation for continuous variables

### Total fluid intake

The estimated total daily fluid (TFI) intakes per age category, sex and country are shown in Table 2. There was no significant sex difference in any of the age groups or countries apart from the Mexican children where boys had a significantly (*P* < 0.039) higher median (25–75th) intake 1155 (809–1540) mL/day compared with girls 994 (719–1452) mL/day. Mexican boys and girls had the lowest median intakes, 994 and 1155 mL/day, respectively; Uruguayan boys had the highest median TFI (1686 mL/day) and Argentinian girls the highest for girls (1594 mL/day). Overall Mexican children and adolescents had the lowest median TFI 1061 (751–1490) compared with the other countries. Uruguayan children and Argentinian adolescents had the highest total fluid intakes.

**Table 2:** Daily total fluid intake (mL/day) for children (4–9 years) and adolescents (10–17 years), by country and sex

	Percentiles										
	Country	Sex	*N* (%)	Mean ± SEM	5	10	25	50	75	90	95
4–9 years	Mexico	M	140 (48)	1301 ± 59	506	581	809	1155^a^	1540	2178	2831
		F	153 (52)	1169 ± 52	441	529	719	994	1452	2092	2493
	Brazil	M	68 (47)	1393 ± 88	495	622	860	1229	1633	2483	3124
		F	78 (53)	1432 ± 72	615	766	1034	1314	1629	2359	2717
	Argentina	M	114 (66)	1843 ± 74	668	903	1203	1665	2414	3006	3401
		F	59 (34)	1737 ± 108	492	875	1098	1594	2228	2872	3481
6–9 years	Uruguay	M	57 (47)	1804 ± 87	931	1106	1316	1686	2014	2885	3345
		F	64 (53)	1594 ± 58	898	1011	1283	1519	1865	2191	2346
	Mexico	M	212 (56)	1687 ± 61	644	758	1052	1469	2121	2923	3521
		F	164 (44)	1669 ± 75	628	737	934	1456	2166	3026	3376
	Brazil	M	62 (32)	1788 ± 142	494	659	1023	1407	2374	3757	4571
		F	132 (68)	1678 ± 76	493	676	1027	1533	2223	2889	3346
	Argentina	M	130 (59)	1932 ± 66	797	966	1383	1876	2442	3020	3194
		F	89 (41)	1845 ± 81	789	971	1335	1710	2291	2626	3323
	Uruguay	M	74 (51)	1745 ± 109	573	865	1170	1587	2176	2793	3668
		F	70 (49)	1587 ± 80	637	770	984	1520	2043	2457	2819
4–9 years	Mexico		293	1232 ± 39	488	562	751	1061	1490	2126	2622
	Brazil		146	1414 ± 56	555	700	957	1297	1632	2359	2678
	Argentina		173	1807 ± 61	632	886	1185	1633	2344	2930	3404
6–9 years	Uruguay		121	1693 ± 52	905	1049	1311	1639	1904	2364	2978
10–17 years	Mexico		376	1679 ± 47	641	747	1011	1462	2121	2988	3393
	Brazil		194	1713 ± 69	497	673	1030	1491	2231	2971	3792
	Argentina		219	1897 ± 51	864	967	1362	1780	2400	2850	3198
	Uruguay		144	1668 ± 68	596	802	1088	1544	2115	2582	3114

*SEM* Standard error of the mean, *M* Males, *F* females

^a^Wilcoxon test was used for sex comparisons *P* < 0.039

### Comparison with IOM recommendations

**Fig. 1:**
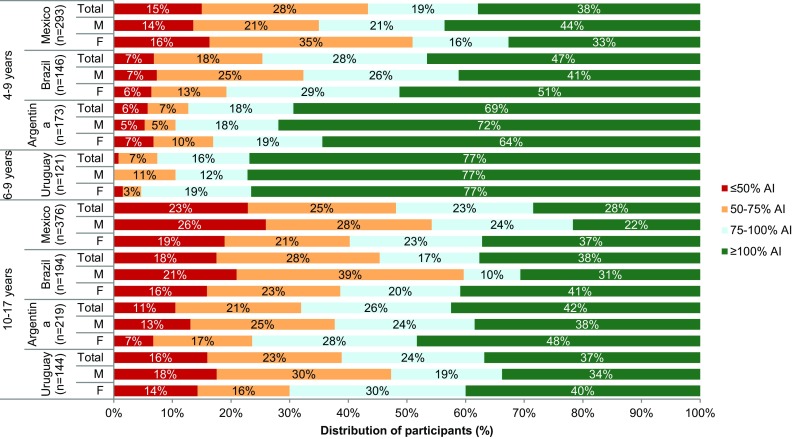
Total fluid intake of children (4–9 years) and adolescents (10–17 years) expressed as a percentage (%) of the adequate intake of water from fluids set by the Institute of Medicine [7] based on 7-day mean of each participant by sex

Figure 1 shows a comparison of TFI with the AI of water from fluids set by the IOM [5]. Over 75% of 4–9-year-old in Uruguay and 64–72% in Argentina met the IOM’s recommended intake. This decreased to 41–50% in Brazil and 33–44% in Mexico. Less than 2% of Uruguayan children drank less than 50% of the AI; this rose to < 7% in Argentina. In Mexico, 16% of boys and 14% girls drank 50% or less of the AI. In the adolescent age group, there was an increase in the number of both males and females who were not drinking 50% of the recommended TFI. Fewer adolescents, than children, in all countries met the AIs for water from fluids. Argentinian females and males, 48 and 38%, respectively, had the highest rates of participants meeting or exceeding the AI. Mexicans again had the lowest rates; 37% for females and 22% for males.

### Type of fluid consumed

The type of fluid consumed by the younger children is shown in Table 3. It is interesting to note that in Brazil all the children drank water, while only 84% of Argentinian children consumed water. In terms of volume of water consumed, Uruguayan and Brazilian children consumed more water than all the other countries; 500 (250–768) and 369 (238–609) mL/day, respectively. In Mexico and Uruguay, the majority of the water consumed was bottled, whereas in Argentina and Brazil it was tap water. The median intakes of tap water were 0 mL/day for Mexican and Uruguayan children and 266 and 138 mL/day for Brazilian and Argentinian children, respectively. Over three quarters of these children consumed milk, or its derivatives, on a daily basis. The highest daily volume consumed was in Uruguay 429 mL/day; Mexico had the lowest milk consumption at 265 mL/day. With the exception of Brazil, where 70% of children consumed 100% fruit juice, this drink was not a popular choice within this age group. Uruguayan children consumed fewer hot beverages than children in the other countries. Only 1% of children in Uruguay drank hot beverages with a median value of 0 mL/day due to the skewed nature of the data. Nearly all children (93–98%) in all four countries consumed SSB; more SSB was consumed than milk and its derivatives. A daily median of more than 500 mL was consumed in Argentina (545 mL) and Uruguay (570 mL). In both these countries, the 75th percentile was greater than 1 L/day. In Mexico and Brazil, the median intake was < 400 mL/day with 75th percentiles of approximately 600 mL/day. In all countries, the major contributor to SSB intake was carbonated soft drinks (CSD), followed by juice-based drinks, which were particularly popular in Brazil.

**Table 3:** Median (P25–P75) daily intake (mL/day) of different fluid types and the percentage of consumers among children (4–9 years), by country

	4–9 years						6–9 years	
	Mexico (*n* = 293)		Brazil (*n* = 146)		Argentina (*n* = 173)		Uruguay (*n* = 121)	
	P50 (P25–P75)	% consumers	P50 (P25–P75)	% consumers	P50 (P25–P75)	% consumers	P50 (P25–P75)	% consumers
Water	252 (90–505)	90	369 (238–609)	100	271 (79–607)	84	500 (250–768)	91
Bottled water	241 (61–468)	87	0 (0–170)	48	0 (0–64)	30	434 (0–706)	73
Tap water	0 (0–0)	16	266 (82–438)	83	138 (0–461)	68	0 (0–50)	26
Milk and derivatives	265 (111–451)	91	302 (152–468)	95	343 (154–517)	87	429 (232–500)	90
Hot beverages	0 (0–28)	33	0 (0–63)	47	54 (0–211)	59	0 (0–0)	1
Coffee	0 (0–0)	23	0 (0–34)	36	0 (0–0)	6	0 (0–0)	0
Tea	0 (0–0)	14	0 (0–0)	17	0 (0–35)	29	0 (0–0)	0
Maté	ND	ND	ND	ND	0 (0–139)	40	0 (0–0)	1
Other hot beverages	ND	ND	ND	ND	ND	ND	0 (0–0)	0
SSB	391 (185–607)	95	396 (197–603)	98	545 (254–1062)	93	570 (275–1026)	98
CSD	48 (0–200)	61	150 (47–267)	88	191 (39–378)	79	217 (200–484)	92
Juice-based drinks	68 (0–216)	67	171 (71–297)	92	161 (0–514)	72	109 (0–595)	55
Functional beverages	0 (0–0)	12	0 (0–0)	13	0 (0–0)	6	0 (0–0)	0
RTD tea and coffee	0 (0–0)	13	0 (0–0)	14	0 (0–0)	3	0 (0–0)	0
Flavored water	57 (0–191)	67	0 (0–0)	14	0 (0–54)	31	0 (0–0)	7
100% fruit juices	0 (0–0)	22	55 (0–158)	70	0 (0–0)	16	0 (0–0)	1
A/NSB	0 (0–0)	11	0 (0–0)	10	0 (0–156)	43	0 (0–0)	5
Alcoholic beverages	0 (0–0)	0	0 (0–0)	0	0 (0–0)	1	0 (0–0)	0
Other beverages	0 (0–0)	9	0 (0–0)	19	0 (0–0)	4	0 (0–0)	17

*SSB* Sugar sweetened beverages, *CSD* carbonated sweetened drinks, *RTD* ready to drink, *A*/*NSB* Artificial/non-nutritive sweeteners beverages, *ND* no data

Types of beverage consumed by the 10–17-year-old are shown in Table 4. Adolescents drank a higher volume of water daily in Argentina, Brazil and Mexico, which was most marked in Brazil and Mexico where the median volume was > 130 mL/day. Brazilian adolescents (10–17 years) had the highest water consumption 505 (278–778) mL/day of any country. As with the younger children, Mexicans and Uruguayans drank mainly bottled water while Argentinian and Brazilian adolescents drank mainly tap water. Uruguay had the lowest consumption of SSB with a median intake of 390 mL/day; CSDs were the major contributor to this volume. The largest consumers of SSB were Argentinian adolescents who had a daily intake of 686 mL/day with a 75th percentile > 1 L; most of this being CSD and juice-based drinks. As might be expected there was a lower intake of milk and its derivatives amongst these older children compared with the younger age groups and a subsequent increase in the intake of hot beverages. As was observed in the children, Brazilian adolescents had the highest median intake of 100% fruit juice (42 mL/day) with 60% consuming it daily.

**Table 4:** Median (P25–P75) daily intake (mL/day) of different fluid types and the percentage of consumers among adolescents (10–17 years), by country

	Mexico (*n* = 376)		Brazil (*n* = 194)		Argentina (*n* = 219)		Uruguay (*n* = 144)	
	P50 (P25–P75)	% consumers	P50 (P25–P75)	% consumers	P50 (P25–P75)	% consumers	P50 (P25–P75)	% consumers
Water	391 (180–963)	90	505 (278–778)	99	346 (93–675)	84	434 (155–798)	89
Bottled water	336 (111–721)	85	17 (0–179)	54	0 (0–100)	38	340 (0–684)	74
Tap water	0 (0–0)	23	314 (114–643)	88	146 (0–497)	68	0 (0–39)	29
Milk and derivatives	154 (9–320)	76	156 (36–265)	83	144 (0–361)	71	240 (63–498)	80
Hot beverages	0 (0–131)	49	31 (0–149)	61	179 (0–357)	75	0 (0–87)	29
Coffee	0 (0–107)	43	16 (0–98)	55	0 (0–73)	35	0 (0–0)	19
Tea	0 (0–0)	17	0 (0–0)	24	0 (0–71)	36	0 (0–0)	8
Maté	ND	ND	ND	ND	0 (0–179)	40	0 (0–0)	13
Other hot beverages	ND	ND	ND	ND	ND	ND	0 (0–0)	1
SSB	524 (258–908)	94	499 (262–811)	98	686 (388–1147)	92	390 (194–749)	92
CSD	137 (0–393)	72	235 (107–459)	93	247 (86–599)	83	200 (64–411)	78
Juice-based drinks	47 (0–200)	58	174 (54–344)	89	150 (0–463)	68	0 (0–215)	42
Functional beverages	0 (0–0)	11	0 (0–0)	15	0 (0–0)	10	0 (0–0)	5
RTD tea and coffee	0 (0–0)	19	0 (0–0)	16	0 (0–0)	1	0 (0–0)	0
Flavored water	115 (0–292)	68	0 (0–0)	17	0 (0–71)	32	0 (0–0)	19
100% fruit juices	0 (0–0)	20	42 (0–157)	60	0 (0–0)	15	0 (0–0)	6
A/NSB	0 (0–0)	12	0 (0–36)	33	0 (0–100)	38	0 (0–0)	18
Alcoholic beverages	0 (0–0)	3	0 (0–0)	7	0 (0–0)	6	0 (0–0)	2
Other beverages	0 (0–0)	10	0 (0–0)	8	0 (0–0)	2%	0 (0–0)	19

*SSB* Sugar sweetened beverages, *CSD* carbonated sweetened drinks, *RTD* ready to drink, *A*/*NSB* Artificial/non-nutritive sweeteners beverages, *ND* no data

**Fig. 2:**
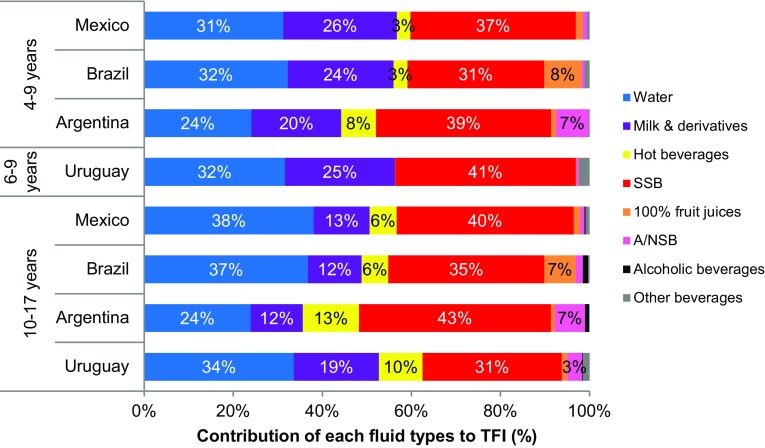
Contribution of the different fluid types (%) to total fluid intake among children (4–9 years) and adolescents (10–17 years), by country. *SSB* Sugar sweetened beverage, *A/NSB* Artificial/non-nutritive sweeteners, *TFI* Total fluid intake

The contribution of each type of fluid to TFI (%) is shown in Fig. 2, and the same data by sex are shown in supplementary figure S3. In all four countries, milk and its derivatives represented about a quarter of TFI in the youngest age group. Children in Mexico and Brazil drank approximately a third of TFI as water and another third as SSB. In Argentina and Uruguay, the largest contributor to TFI was SSB. Amongst adolescents, milk and its derivatives contributed to 12–19% of TFI. The contribution of CSD and water to TFI differed in the adolescents compared with the children. In Argentina SSB contributed 43% to TFI compared with 24% for water. In the other three countries, the contribution of SSB and water was similar, namely, 38 vs 40% in Mexico, 37 vs 35% in Brazil and 34 vs 31% in Uruguay.

**Fig. 3:**
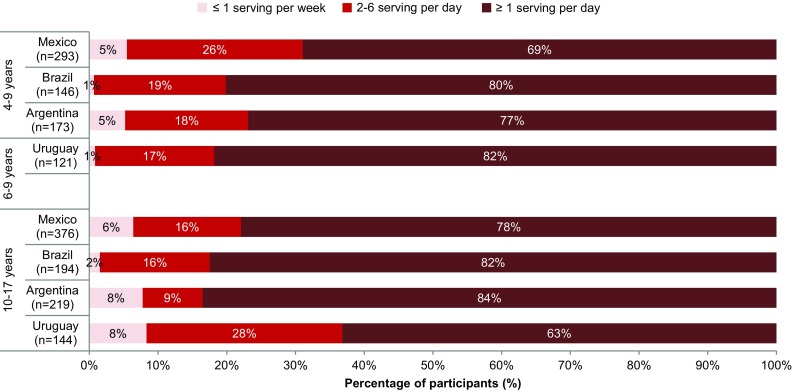
Percentage of children and adolescents drinking SSB on daily or less frequency, according to country

Over 75% of children and adolescents in Mexico, Brazil and Argentina drank at least one serving (250 mL/day) of SSB per day (Fig. 3). In Mexico, Brazil and Argentina, the number of children drinking ≥ 1 serving per day was slightly lower than for the corresponding adolescents. However, Uruguay had the most children and the lowest number of adolescents drinking ≥ 1 serving per day.

## Discussion

The present study reports recent data for children (aged 4–9 years) and adolescents (aged 10–17 years) on estimated total fluid intake and types of fluid consumed in four Latin American countries. For the first time, comparisons with the IOM recommendations [5] on adequate intake (AI) of water from fluids (20% of TWI) are reported for children and adolescents in Argentina, Brazil and Uruguay. It also presents more recent data for these age groups in Mexico. In the present study, Mexican children had a lower median TFI than children in the other countries (1061 mL/day); Uruguayan children had the highest daily intake of fluid at 1639 mL/day. Amongst adolescents, Mexico, Uruguay and Brazil had similar TFIs (1462–1544 mL/day); Argentinian adolescents had the highest TFI (1780 mL/day). It is difficult to compare the results of the present survey with most of the previous studies due to different age categories and methodological differences. The present study generally reported fluid intakes higher than from the latest data reported from Mexico in 2012 [4] but lower than those from Brazil [8] from 2008/9. No data are available for children and adolescents from Argentina or Uruguay apart from the in *Liq.In*^*7*^ surveys of 2012 [6]. Comparisons with the previous *Liq.In*^*7*^ study [6] are more appropriate as the same validated 7-day record and age categories were used. Intake by Mexican and Brazilian children and Brazilian adolescents in the present survey were similar to the earlier study; Argentinian children and Mexican and Argentinian adolescents increased their TFI by approximately 260 mL/day. However, the TFIs for Uruguayan children and adolescents decreased markedly by approximately 300 and 500 mL/day, respectively. The reasons for the TFI differences between the two surveys are unclear although it may be due to sampling differences, despite the populations being selected according to the same quota method. In particular, the sample size for Uruguay doubled for children and increased by nearly 50% for the adolescents.

Over two-thirds of children in Argentina and Uruguay met the AIs [5], although in Mexico and Brazil fewer than half of children met them. These rates are broadly comparable to the previous *Liq.In*^*7*^ study [6] for Mexico, Uruguay and Argentina; however, the percentage of Brazilian children not meeting the recommendations increased from 32% to over 50% when either the IOM [5] or the EFSA [14] recommendations were used. It has previously been shown that children and adolescents frequently do not meet to recommendations on adequate water intake of TFI [20–22] with up to 90% drinking less than the recommendations in some countries. While it is reassuring to note that a reasonable proportion of children and adolescents in these four Latin American countries appear to have adequate intakes, there is still concern about the health and well-being of those children who report intakes below the recommendation. While without biomarkers it is not possible to draw conclusions about their hydration status, if their water intake is actually suboptimal it is possible that their cognitive and physical performances may be affected [1, 3].

An important issue that has emerged from the present study is that while these children and adolescents are more likely to meet the recommendations on water intake from fluids, a significant proportion of the fluid was sweetened beverages, especially CSD. This was consistent for all countries but most marked in Uruguay. In a period of 4 years, Uruguayan children increased their proportion of TFI from SSB from 25% in the earlier *Liq.In*^*7*^ study [23] to 41% in the present study. Both Uruguayan and Argentinian adolescents also appear to have increased the proportion of fluid from SSB during this period. This comparison, however, should be made with caution due to differing classifications of fruit juice between the studies. There is a tendency to snack in Latin American countries [24] with SSB being a snack component amongst younger adults and children [25]. It is interesting to note that the methodology used in this study, and the previous *Liq.In*^*7*^ studies [6, 23] is more likely to have captured all drinking events including snacks [26] and, therefore, may better reflect TFI and SSB intake.

These levels of SSB consumption raise concerns given the increasing body of evidence on the negative effects of some drinks on children’s health [13]. The apparent increase in SSB consumption between the previous and present *Liq.In*^*7*^ studies was most marked in Uruguayan children and adolescents, and Argentinian adolescents. Latin American has seen a rapid rise in obesity and overweight especially in children and adolescents [12, 27] and more public health policies are urgently needed to halt, and hopefully, reverse this trend. In Mexico, several policies have been implemented including a tax on SSB since 2014 [28] and the development of a healthy beverage guide [29]. While beyond the remit of this analysis, comparison between countries, stratified for BMI status and socioeconomic status may yield further insights into drinking behavior amongst children and adolescents in Latin America. Data from the present study are vital in highlighting high consumption levels of different fluid types and for the development of health policies. This is particularly important in a country such as Uruguay where fluid consumption data are lacking. Uruguay, like other countries in this region, is also experiencing the double burden of disease with undernutrition occurring alongside obesity [30], which will further stretch limited public health resources.

One strategy that is increasingly being used to reduce SBB consumption in children is to increase plain water consumption [31]. Studies have shown that higher consumption of plain water is associated with lower consumption of SSB [32–34]. In the present study, the median intake of water ranged from 252 to 500 mL/day, with the Uruguayan children drinking more than the other groups of children; Mexican children drank the lowest volume of water. As a proportion of TFI in children, water accounted for 24% in Argentina and 31–32% in the other three countries. The adolescents had a similar pattern when proportion of TFI was considered. These results are broadly similar to the previous *Liq.In*^*7*^ surveys for Mexico, Argentina and Brazil. Although Uruguayan children and adolescents had markedly lower water intakes in this study than in the previous survey [6], this was accompanied by an increase in SSB consumption, as discussed above. This finding suggests that action is needed to reverse this trend in Uruguay. Plain water is the drink of choice and healthy hydration strategies should be incorporated into public health policies and food-based dietary guidelines [35]. An example of this is the Mexico healthy beverage guide, which includes a pictorial representation of a jug that represents daily fluid intake and shows the proportion of each fluid type that should be consumed [29].

The strengths of this study include the use of a methodology validated, albeit in adults, to assess fluid intake, namely, the *Liq.In*^*7*^ dairy [16]. This methodology was previously used by Iglesia [6] in the same Latin American countries, so facilitating valid temporal comparisons. The large sample size also contributed to a fuller understanding of fluid intake in these countries. Nonetheless, there are limitations to this study including those inherent in any cross-sectional study, such as how representative were the samples of the general populations in these age categories. In this study, a quota sampling system was used in terms of age, sex and SES. Data from Brazilian children and adolescents were collected from only one city, while it is the largest city and municipality in Brazil, it may not be representative of other areas of Brazil. Moreover, data collection in Brazil was performed during another a different period of the year for operational reasons. As all data collection was performed outside summer or winter, periods with larger temperature variations, the seasonal effect on fluid intake behavior was considered to be moderate. Recording dietary intake in children requires a degree of parental assistance according to age [36] and for all children under 12 years a parent or care giver  completed the 7-day record. The validity of data of this age group, and adolescents, remains to be assessed. Due to operational restrictions, 4- and 5-year-old were not included in the Uruguayan sample, which may have skewed the data slightly, but only accounted for 6% of the overall total of the age group. This survey aimed to describe fluid intake behavior, not TWI; therefore, data on food moisture were not collected and any conclusions on TWI must be drawn with care. Similarly, hydration biomarkers were not used and, therefore, no conclusions can be drawn as to the hydration status of this population.

## Conclusions

The cross-sectional study reported here shows data on TFI, type of fluid consumed and adherence to AI recommendations for water from fluids [5] for Argentina, Brazil, Mexico and Uruguay. A sizeable proportion of children and adolescents in these four Latin American countries reported intakes close to the recommendations. However, many of the younger children and more than half of the adolescents in this sample did not meet the recommendations. Such intakes have been associated with an increased risk of hypohydration. The amount of SSB consumed in 2016 appears to have increased since the previous *Liq.In*^*7*^ study conducted in 2012. Additionally, the proportion of children drinking one serving of SSB daily is a reason for concern, given the negative effects of SSB consumption on children’s health. These observations provide valuable information to support current and future public policies and programs, aimed at installing healthier drinking habits in children and adolescents in the Latin American region.

## Electronic supplementary material

Below is the link to the electronic supplementary material.

Supplementary material 1 (DOCX 111 KB)
