# Supplementary material for: Fluid intake of Latin American children and adolescents: results of four 2016 LIQ.IN7 National Cross-Sectional Surveys
Source: Eur J Nutr. 2018 Jun 1;57(Suppl 3):53–63. doi: 10.1007/s00394-018-1728-8 (PMC6008370; doi:10.1007/s00394-018-1728-8)
Supplement: Supplementary file 1 — Supplementary material 1 (DOCX 111 KB) [file 394_2018_1728_MOESM1_ESM.docx]

**SUPPLEMENTARY MATERIALS**

**FLUID INTAKE OF LATIN AMERICAN CHILDREN AND ADOLESCENTS: RESULTS OF FOUR 2016 LIQ.IN^7^ NATIONAL CROSS-SECTIONAL SURVEYS**

*Gandy J,^1,2*^ Martinez H, ^3^ Carmuega E,^4^ Arredondo JL,^5^ Pimentel C,^5^ Moreno LA,^6,7^ Kavouras SA,^8,9^ Salas-Salvadó J, ^7,10^*

^1^ British Dietetic Association, Birmingham, UK.

^2^ School of Life and Medical services, University of Hertfordshire, Hatfield, UK.

^3^ Hospital Infantil de México Federico Gómez, México City, México.

^4^ Center of Studies on Infant Nutrition, Buenos Aires, Argentina.

^5^ Unidad de Apoyo a la Investigación Clínica, Instituto Nacional de Pediatría, Mexico City, Mexico.

^6^ GENUD (Growth, Exercise, NUtrition and Development) Research Group, Faculty of Health Sciences, Universidad de Zaragoza, Instituto Agroalimentario de Aragón (IA2), Instituto de Investigación Sanitaria Aragón (IIS Aragón), Zaragoza, Spain.

^7^ CIBERobn (Centro de Investigación Biomédica en Red Fisiopatología de la Obesidad y Nutrición), Institute of Health Carlos III, Madrid, Spain.

^8^ Hydration Science Lab, University of Arkansas, Fayetteville, AR, USA.

^9^ Division of Endocrinology, University of Arkansas for Medical Sciences, Little Rock, AR, USA.

^10^ Human Nutrition Unit, Hospital Universitari de Sant Joan de Reus, Faculty of Medicine and Health Sciences, Institut d’Investigació Sanitària Pere Virgili, Biochemistry and Biotechnology Department, Universitat Rovira i Virgili. C/ Sant Llorenç, 21, 43201 Reus (Spain).

*** Corresponding author:** Joan GANDY, School of Life and Medical services, University of Hertfordshire, Hatfield, AL10 9AB, UK. joan.gandy@btinternet.com

**Table S1** Classification of the fluid types

| Classification of fluids | Detailed Fluid types |
| --- | --- |
| **Water** |  |
| *Bottled water* | Unflavored still water, unflavored sparkling water |
| *Tap water* | Tap water, filtered water, boiled water |
| **Milk & derivatives** | Low fat and full fat milk, raw milk, ready-to-drink flavored milk, homemade flavored milk, yogurt milk, atole/champurrado, powder milk, powder/syrup flavored milk, fruit shake with milk, cocoa compound with milk, vegetal milk (Soya, almond…) |
| **Hot beverages** |  |
| *Coffee* | Coffee, coffee from coffee maker, powder instant coffee, vending machine coffee, restaurant/franchise coffee |
| *Tea* | Homemade hot/cold tea (from tea bags), infusions (herbal), restaurant/franchise tea |
| *Maté* | Mate, mate cocido |
| *Other hot beverages* | Hot Beverages other than coffee, tea or maté. For example *Amargos (Uruguay)* |
| **SSB** |  |
| *Carbonated sweet drinks* (*CSD)* | Cola regular, Concentrated/powder juice with sparkling water, flavored water sparkling, fruit flavored sparkling |
| *Juice-based drinks* | Eskimo/smoothies (ready to drink or homemade), packaged fruit juice/nectar/vegetable, fruit shake with water, powder/syrups water, still lemonade |
| *Functional beverages* | Sports drinks, tonic regular, energy drinks, flavored water enriched with vitamins/minerals, quina water |
| *RTD Tea & Coffee* | Bottled coffee, Ice coffee, ready to drink bottled tea, ready to drink/homemade ice tea, powder tea |
| *Flavored water* | Flavored packaged/homemade water, *aguas frescas,* coconut water |
|  |  |
| **100% fruit juices** | Natural juice (Vegetable/fruit), bottled 100% fruit juice & vegetables juice, homemade freshly squeezed juice, ready to drink freshly squeezed juice, freshly squeezed juice "take from outside" |
|  |  |
| **A/NSB** | Cola light/zero, flavored water zero/light, fruit flavored light, light bottled tonic, light bottled ice tea, bottled juice light |
|  |  |
| **Alcoholic beverages** | Beer, cocktails, pure whiskey/vodka/gin, spirit/liquor/digestive, wine, champagne, cider |
|  |  |
| **Other beverages** | Beverages identified by participants as “other than listed above”, packaged soy drinks, agua de arroz (Mexico), diet drinks as meal replacement (slim fast), ready to drink soy based juice, Beer 0% Alcohol |

*SSB* sugar sweetened beverages, *A/NSB* Artifical/non nutritive sweeteners beverages

**Table S2a** Median (P25-P75) daily intake (mL/day) of different fluid types and the percentage of consumers in Mexico by sex and age categories

|  | 4-9 years | | | | 10-17 years | | | |
| --- | --- | --- | --- | --- | --- | --- | --- | --- |
|  | Males (n=140) | | Females (n=153) | | Males (n=212) | | Females (n=164) | |
|  | **P50 (P25-P75)** | **%**  **consumers** | **P50 (P25-P75)** | **% consumers** | **P50 (P25-P75)** | **%**  **consumers** | **P50 (P25-P75)** | **%**  **consumers** |
| Water | 251 (102-566) | 94 | 257 (79-459) | 86 | 399 (173-1010) | 89 | 386 (188-935) | 92 |
| *Bottled water* | *240 (65-543)* | *91* | *243 (45-439)* | *83* | *317 (123-709)* | *83* | *354 (85-837)* | *88* |
| *Tap water* | *0 (0-0)* | *17* | *0 (0-0)* | *14* | *0 (0-0)* | *24* | *0 (0-0)* | *21* |
| Milk & derivatives | 325 (122-486) | 93 | 232 (96-368) | 90 | 171 (15-324) | 77 | 135 (1-285) | 75 |
| Hot beverages | 0 (0-57) | 36 | 0 (0-18) | 29 | 0 (0-143) | 50 | 0 (0-126) | 49 |
| *Coffee* | *0 (0-16)* | *27* | *0 (0-0)* | *20* | *0 (0-114)* | *42* | *0 (0-103)* | *43* |
| *Tea* | *0 (0-0)* | *16* | *0 (0-0)* | *13* | *0 (0-0)* | *16* | *0 (0-0)* | *19* |
| *Mate* | *ND* | *ND* | *ND* | *ND* | *ND* | *ND* | *ND* | *ND* |
| *Other hot beverages* | *ND* | *ND* | *ND* | *ND* | *ND* | *ND* | *ND* | *ND* |
| SSB | 394 (183-588) | *95* | 387 (190-627) | *95* | 516 (252-909) | *93* | 542 (261-902) | *95* |
| *CSD* | *42(0-196)* | *64* | *51 (0-205)* | *59* | *146 (0-405)* | *73* | *133 (0-352)* | *71* |
| *Juice-based drinks* | *57 (0-175)* | 63 | *71 (0-235)* | 71 | *43 (0-188)* | 56 | *62 (0-244)* | 60 |
| *Functional beverages* | *0 (0-0)* | *16* | *0 (0-0)* | *8* | *0 (0-0)* | *11* | *0 (0-0)* | *10* |
| *RTD tea & coffee* | *0 (0-0)* | *14* | *0 (0-0)* | *12* | *0 (0-0)* | *19* | *0 (0-0)* | *20* |
| *Flavored water* | *64 (0-230)* | *69* | *54 (0-181)* | *65* | *97 (0-263)* | *67* | *143 (0-324)* | *70* |
| 100% fruit juices | *0 (0-0)* | *19* | *0 (0-0)* | *24* | *0 (0-0)* | *20* | *0 (0-0)* | *21* |
| A/NSB | *0 (0-0)* | *9* | *0 (0-0)* | *14* | *0 (0-0)* | *12* | *0 (0-0)* | *12* |
| Alcoholic beverages | *0 (0-0)* | 0 | *0 (0-0)* | 0 | *0 (0-0)* | 3 | *0 (0-0)* | 3 |
| Other beverages | *0 (0-0)* | 9 | *0 (0-0)* | 8 | *0 (0-0)* | 9 | *0 (0-0)* | 10 |

*SSB* sugar sweetened beverages, *CSD* carbonated sweetened drinks, *RTD* ready to drink, *A/NSB* artificial/non-nutritive sweeteners beverages, *ND* no data.

**Table S2b** Median (P25-P75) daily intake (mL/day) of different fluid types and the percentage of consumers in Brazil by sex and age category

|  | 4-9 years | | | | 10-17 years | | | |
| --- | --- | --- | --- | --- | --- | --- | --- | --- |
|  | Males (n=68) | | Females (n=78) | | Males (n=62) | | Females (n=132) | |
|  | **P50 (P25-P75)** | **%**  **consumers** | **P50 (P25-P75)** | **%**  **consumers** | **P50 (P25-P75)** | **%**  **consumers** | **P50 (P25-P75)** | **% consumers** |
| Water | 347 (216-626) | 100 | 405 (265-603) | 100 | 571 (273-849) | 98 | 463 (279-757) | 100 |
| *Bottled water* | *0 (0-119)* | *41* | *36 (0-291)* | *54* | *8 (0-101)* | *52* | *20 (0-245)* | *55* |
| *Tap water* | *266 (84-458)* | *85* | *257 (64-429)* | *81* | *398 (160-755)* | *87* | *287 (107-589)* | *88* |
| Milk & derivatives | 307 (180-477) | 99 | 296 (113-456) | 91 | 181 (77-308) | 85 | 135 (30-253) | 82 |
| Hot beverages | 8 (0-79) | 53 | 0 (0-36) | 42 | 20 (0-104) | 58 | 35 (0-157) | 63 |
| *Coffee* | *0 (0-59)* | *43* | *0 (0-20)* | *29* | *7 (0-97)* | *52* | *25 (0-103)* | *57* |
| *Tea* | *0 (0-0)* | *13* | *0 (0-0)* | *21* | *0 (0-0)* | *19* | *0 (0-12)* | *27* |
| *Mate* | *ND* | *ND* | *ND* | *ND* | *ND* | *ND* | *ND* | *ND* |
| *Other hot beverages* | *ND* | *ND* | *ND* | *ND* | *ND* | *ND* | *ND* | *ND* |
| SSB | 424 (159-552) | *99* | 372 (227-625) | 97 | 458 (244-795) | *95* | 532 (288-812) | *100* |
| *CSD* | *133 (59-248)* | *87* | *154 (44-288)* | *88* | 238 *(87-433)* | *92* | *234 (112-464)* | *94* |
| *Juice-based drinks* | *166 (43-373)* | 88 | *175 (85-285)* | *95* | 162 *(57-333)* | 89 | *176 (52-354)* | 89 |
| *Functional beverages* | *0 (0-0)* | *13* | *0 (0-0)* | *13* | *0 (0-0)* | *16* | *0 (0-0)* | *15* |
| *RTD tea & coffee* | *0(0-0)* | *4* | *0 (0-0)* | *23* | *0 (0-0)* | *13* | *0 (0-0)* | *18* |
| *Flavored water* | *0 (0-0)* | *13* | *0 (0-0)* | *15* | *0 (0-0)* | *21* | *0 (0-0)* | *15* |
| 100% fruit juices | 36 (0-119) | *66* | 71 (0-177) | 73 | 68 (0-155) | *66* | 32 (0-157) | *58* |
| A/NSB | 0 (0-0) | *7* | *0 (0-0)* | 12 | 0 (0-30) | *31* | 0 (0-36) | *34* |
| Alcoholic beverages | 0 (0-0) | 0 | *0 (0-0)* | 0 | *0 (0-0)* | 2 | 0 (0-0) | 9 |
| Other beverages | 0 (0-5) | 25 | *0 (0-0)* | 14 | *0 (0-0)* | 10 | 0 (0-0) | 8 |

*SSB* sugar sweetened beverages, *CSD* carbonated sweetened drinks, *RTD* ready to drink, *A/NSB* artificial/non-nutritive sweeteners beverages, *ND* no data

**Table S2c** Median (P25-P75) daily intake (mL/day) of different fluid types and the percentage of consumers in Argentina by sex and age category

|  | 4-9 years | | | | 10-17 years | | | |
| --- | --- | --- | --- | --- | --- | --- | --- | --- |
|  | Males (n=114) | | Females (n=59) | | Males (n=130) | | Females (n=89) | |
|  | **P50 (P25-P75)** | **%**  **consumers** | **P50 (P25-P75)** | **%**  **consumers** | **P50 (P25-P75)** | **%**  **consumers** | **P50 (P25-P75)** | **%**  **consumers** |
| Water | 300 (85-641) | 86 | 243 (61-480) | 81 | 250 (35-645) | 79 | 409 (186-722) | 91 |
| *Bottled water* | *0 (0-3)* | *25* | *0 (0-154)* | *41* | *0 (0-72)* | *34* | *0 (0-124)* | *44* |
| *Tap water* | *168 (0-528)* | *75* | *60 (0-321)* | *54* | *110 (0-466)* | *64* | *214 (0-549)* | *74* |
| Milk & derivatives | 357 (149-506) | 87 | 336 (179-536) | 88 | 154 (0-405) | 69 | 125 (0-286) | 74 |
| Hot beverages | 73 (0-214) | 59 | 53 (0-193) | 59 | 161 (0-333) | 72 | 214 (52-412) | 80 |
| *Coffee* | *0(0-0)* | *8* | *0(0-0)* | *3* | *0 (0-47)* | *28* | *0 (0-114)* | *46* |
| *Tea* | *0 (0-36)* | *31* | *0 (0-34)* | *27* | *0 (0-51)* | *29* | *0 (0-107)* | *46* |
| *Mate* | *0 (0-100)* | *38* | *0 (0-188)* | *44* | *0 (0-179)* | *41* | *0 (0-154)* | *38* |
| *Other hot beverages* | *ND* | *ND* | *ND* | *ND* | *ND* | *ND* | *ND* | *ND* |
| SSB | 590 (271-1111) | 94 | 532 (206-817) | 92 | 854 (442-1348) | 92 | 611 (328-843) | 92 |
| *CSD* | *194 (61-367)* | *83* | *175 (0-406)* | *69* | *340 (102-645)* | *85* | *224 (47-526)* | *79* |
| *Juice-based drinks* | *177 (43-631)* | *78* | *137 (0-394)* | *61* | *102 (0-519)* | *65* | *186 (0-422)* | *73* |
| *Functional beverages* | *0(0-0)* | *4* | *0(0-0)* | *8* | *0(0-0)* | *9* | *0(0-0)* | *11* |
| *RTD tea & coffee* | *0(0-0)* | *1* | *0(0-0)* | *7* | *0(0-0)* | *2* | *0(0-0)* | *1* |
| *Flavored water* | *0 (0-60)* | *32* | *0 (0-54)* | *31* | *0 (0-91)* | *37* | *0 (0-9)* | *26* |
| 100% fruit juices | 0 (0-0) | 15 | 0(0-0) | 19 | *0(0-0)* | 14 | *0(0-0)* | 16 |
| A/NSB | 0 (0-180) | 42 | 0 (0-88) | 44 | 0 (0-84) | 37 | 0 (0-131) | 40 |
| Alcoholic beverages | 0(0-0) | 1 | *0(0-0)* | 2 | 0(0-0) | 8 | 0(0-0) | 3 |
| Other beverages | 0(0-0) | 5 | *0(0-0)* | 2 | 0(0-0) | 1 | 0(0-0) | 3 |

*SSB* sugar sweetened beverages, *CSD* carbonated sweetened drinks, *RTD* ready to drink, *A/NSB* artificial/non-nutritive sweeteners beverages, *ND* no data

**Table S2d** Median (P25-P75) daily intake (mL/day) of different fluid types and the percentage of consumers in Uruguay by sex and age category

|  | 4-9 years | | | | 10-17 years | | | | | |  |
| --- | --- | --- | --- | --- | --- | --- | --- | --- | --- | --- | --- |
|  | Males (n=57) | | Females (n=64) | | Males (n=74) | | Females (n=70) | | | |  |
|  | P50 (P25-P75) | %  consumers | P50 (P25-P75) | %  consumers | P50 (P25-P75) | %  consumers | | P50 (P25-P75) | %  consumers |  |  |
| Water | 586 (246-768) | 93 | 493 (250-770) | 89 | 431 (137-764) | 89 | | 450 (165-875) | 89 |  |  |
| *Bottled water* | *450 (11-736)* | *75* | *418 (0-706)* | *70* | *338 (12-664)* | *77* | | *340 (0-694)* | *71* |  |  |
| *Tap water* | *0 (0-104)* | *30* | *0 (0-0)* | *23* | *0 (0-0)* | *22* | | *0 (0-113)* | *37* |  |  |
| Milk & derivatives | 429 (232-500) | 88 | 390 (223-500) | 92 | 250 (82-519) | 81 | | 218 (33-458) | 79 |  |  |
| Hot beverages | 0 (0-0) | 0 | 0 (0-0) | 2 | 0 (0-82) | 28 | | 0 (0-133) | 30 |  |  |
| *Coffee* | *0 (0-0)* | *0* | *0 (0-0)* | *0* | *0 (0-0)* | *18* | | *0 (0-0)* | *21* |  |  |
| *Tea* | *0 (0-0)* | *0* | *0 (0-0)* | *0* | *0 (0-0)* | *7* | | *0 (0-0)* | *9* |  |  |
| *Mate* | *0 (0-0)* | *0* | *0 (0-0)* | *2* | *0 (0-0)* | *11* | | *0 (0-0)* | *14* |  |  |
| *Other hot beverages* | *0 (0-0)* | *0* | *0 (0-0)* | *0* | *0 (0-0)* | *1* | | *0 (0-0)* | *0* |  |  |
| SSB | 593 (291-1075) | 98 | 539 (266-946) | 98 | 439 (199-936) | 97 | | 355 (166-698) | 87 |  |  |
| *CSD* | *217(150-525)* | *89* | *217 (200-450)* | *94* | *217 (81-434)* | *84* | | *196 (0-363)* | *73* |  |  |
| *Juice-based drinks* | *137 (0-519)* | *56* | *100 (0-596)* | *55* | *0 (0-274)* | *47* | | *0 (0-160)* | *37* |  |  |
| *Functional beverages* | *0 (0-0)* | *0* | *0 (0-0)* | *0* | *0 (0-0)* | *7* | | *0 (0-0)* | *3* |  |  |
| *RTD tea & coffee* | *0 (0-0)* | *0* | *0 (0-0)* | *0* | *0 (0-0)* | *0* | | *0 (0-0)* | *0* |  |  |
| *Flavored water* | *0 (0-0)* | *7* | *0 (0-0)* | *8* | *0 (0-0)* | *23* | | *0 (0-0)* | *14* |  |  |
| 100% fruit juices | 0 (0-0) | 2 | 0 (0-0) | 0 | 0 (0-0) | 7 | | 0 (0-0) | 6 |  |  |
| A/NSB | 0 (0-0) | 4 | 0 (0-0) | 6 | 0 (0-0) | 15 | | 0 (0-0) | 21 |  |  |
| Alcoholic beverages | 0 (0-0) | 0 | 0 (0-0) | 0 | 0 (0-0) | 3 | | 0 (0-0) | 1 |  |  |
| Other beverages | 0 (0-0) | 14 | 0 (0-0) | 19 | 0 (0-0) | 22 | | 0 (0-0) | 16 |  |  |

*SSB* sugar sweetened beverages, *CSD* carbonated sweetened drinks, *RTD* ready to drink, *A/NSB* artificial/non-nutritive sweeteners beverages, *ND* no data

**Table S3a** Mean daily intake (SEM) of the different fluid types (mL/day) in Mexico by sex and age category

|  | 4-9 years | | | | | | 10-17 years | | | | | |
| --- | --- | --- | --- | --- | --- | --- | --- | --- | --- | --- | --- | --- |
|  | Total | | Males | | Females | | Total | | Males | | Females | |
| **TFI** | **1232** | **±39** | **1301** | **±59** | **1169** | **±52** | **1679** | **±47** | **1687** | **±61** | **1669** | **±75** |
| Water | 384 | ±26 | 394 | ±35 | 375 | ±38 | 639 | ±35 | 663 | ±49 | 607 | ±48 |
| *Bottled water* | 362 | ±26 | 373 | ±35 | 352 | ±37 | 552 | ±33 | 557 | ±46 | 545 | ±47 |
| *Tap water* | 23 | ±7 | 22 | ±8 | 23 | ±11 | 87 | ±15 | 105 | ±23 | 62 | ±17 |
| Milk & derivatives | 315 | ±16 | 359 | ±24 | 274 | ±20 | 211 | ±12 | 220 | ±16 | 201 | ±18 |
| Hot beverages | 37 | ±6 | 47 | ±10 | 28 | ±7 | 102 | ±10 | 103 | ±13 | 101 | ±15 |
| *Coffee* | 22 | ±5 | 29 | ±8 | 17 | ±6 | 84 | ±9 | 89 | ±12 | 77 | ±13 |
| *Tea* | 15 | ±3 | 18 | ±5 | 12 | ±4 | 18 | ±4 | 14 | ±3 | 24 | ±7 |
| *Mate* | 0 | ±0 | 0 | ±0 | 0 | ±0 | 0 | ±0 | 0 | ±0 | 0 | ±0 |
| *Other hot beverages* | 0 | ±0 | 0 | ±0 | 0 | ±0 | 0 | ±0 | 0 | ±0 | 0 | ±0 |
| SSB | 458 | ±23 | 464 | ±36 | 452 | ±30 | 667 | ±29 | 634 | ±34 | 710 | ±50 |
| *CSD* | 125 | ±11 | 120 | ±14 | 131 | ±17 | 266 | ±19 | 265 | ±24 | 267 | ±31 |
| *Juice-based drinks* | 155 | ±13 | 139 | ±19 | 170 | ±19 | 160 | ±15 | 150 | ±18 | 172 | ±25 |
| *Functional beverages* | 18 | ±6 | 25 | ±9 | 12 | ±8 | 14 | ±3 | 15 | ±4 | 13 | ±4 |
| *RTD tea & coffee* | 11 | ±2 | 11 | ±3 | 11 | ±3 | 25 | ±4 | 26 | ±5 | 25 | ±6 |
| *Flavored water* | 148 | ±13 | 169 | ±21 | 129 | ±15 | 202 | ±13 | 179 | ±16 | 233 | ±23 |
| 100% fruit juices | 19 | ±3 | 18 | ±4 | 21 | ±5 | 25 | ±4 | 28 | ±6 | 22 | ±5 |
| A/NSD | 11 | ±3 | 8 | ±3 | 15 | ±6 | 15 | ±3 | 16 | ±4 | 15 | ±4 |
| Alcoholic beverages | 0 | ±0 | 0 | ±0 | 0 | ±0 | 4 | ±2 | 5 | ±3 | 4 | ±2 |
| Other beverages | 7 | ±3 | 12 | ±6 | 3 | ±1 | 15 | ±4 | 18 | ±6 | 10 | ±3 |

*TFI* total fluid intake*, SSB* sugar sweetened beverages, *CSD* carbonated sweetened drinks, *RTD* ready to drink, *A/NSB* artificial/non-nutritive sweeteners beverages, *ND* no data

**Table S3b** Mean daily intake (SEM) of the different fluid types (mL/day) in Brazil by sex and age category

|  | 4-9 years | | | | | | 10-17 years | | | | | |
| --- | --- | --- | --- | --- | --- | --- | --- | --- | --- | --- | --- | --- |
|  | Total | | Males | | Females | | Total | | Males | | Females | |
| **TFI** | **1414** | **±56** | **1393** | **±88** | **1432** | **±72** | **1713** | **±69** | **1788** | **±142** | **1678** | **±76** |
| Water | 455 | ±26 | 442 | ±39 | 467 | ±35 | 630 | ±41 | 667 | ±69 | 612 | ±50 |
| *Bottled water* | 130 | ±18 | 93 | ±21 | 163 | ±28 | 182 | ±32 | 145 | ±43 | 199 | ±43 |
| *Tap water* | 325 | ±27 | 349 | ±41 | 305 | ±35 | 448 | ±34 | 522 | ±66 | 413 | ±39 |
| Milk & derivatives | 336 | ±21 | 365 | ±32 | 310 | ±26 | 207 | ±17 | 237 | ±30 | 193 | ±20 |
| Hot beverages | 44 | ±6 | 52 | ±9 | 38 | ±8 | 102 | ±11 | 81 | ±15 | 113 | ±15 |
| *Coffee* | 29 | ±5 | 35 | ±7 | 24 | ±6 | 75 | ±9 | 69 | ±14 | 78 | ±11 |
| *Tea* | 15 | ±4 | 16 | ±7 | 13 | ±4 | 27 | ±7 | 11 | ±4 | 35 | ±11 |
| *Mate* | 0 | ±0 | 0 | ±0 | 0 | ±0 | 0 | ±0 | 0 | ±0 | 0 | ±0 |
| *Other hot beverages* | 0 | ±0 | 0 | ±0 | 0 | ±0 | 0 | ±0 | 0 | ±0 | 0 | ±0 |
| SSB | 434 | ±25 | 426 | ±40 | 441 | ±33 | 600 | ±32 | 611 | ±67 | 595 | ±36 |
| *CSD* | 184 | ±14 | 174 | ±19 | 192 | ±20 | 316 | ±21 | 325 | ±45 | 312 | ±23 |
| *Juice-based drinks* | 225 | ±17 | 227 | ±27 | 222 | ±23 | 237 | ±17 | 240 | ±32 | 235 | ±21 |
| *Functional beverages* | 9 | ±3 | 11 | ±5 | 6 | ±2 | 16 | ±4 | 21 | ±11 | 13 | ±3 |
| *RTD tea & coffee* | 9 | ±3 | 4 | ±2 | 14 | ±6 | 11 | ±2 | 5 | ±2 | 14 | ±3 |
| *Flavored water* | 8 | ±2 | 9 | ±4 | 6 | ±2 | 20 | ±5 | 19 | ±9 | 21 | ±6 |
| 100% fruit juices | 120 | ±22 | 82 | ±13 | 153 | ±39 | 121 | ±17 | 147 | ±45 | 108 | ±15 |
| A/NSD | 9 | ±3 | 11 | ±7 | 8 | ±3 | 28 | ±5 | 29 | ±11 | 27 | ±4 |
| Alcoholic beverages | 0 | ±0 | 0 | ±0 | 0 | ±0 | 21 | ±7 | 12 | ±12 | 25 | ±8 |
| Other beverages | 15 | ±4 | 16 | ±6 | 15 | ±6 | 5 | ±2 | 5 | ±3 | 5 | ±2 |

*TFI* total fluid intake*, SSB* sugar sweetened beverages, *CSD* carbonated sweetened drinks, *RTD* ready to drink, *A/NSB* artificial/non-nutritive sweeteners beverages, *ND* no data

**Table S3c** Mean daily intake (SEM) of the different fluid types (mL/day) in Argentina by sex and age category

|  | 4-9 years | | | | | | 10-17 years | | | | | |
| --- | --- | --- | --- | --- | --- | --- | --- | --- | --- | --- | --- | --- |
|  | Total | | Males | | Females | | Total | | Males | | Females | |
| **TFI** | **1807** | **±61** | **1843** | **±74** | **1737** | **±108** | **1897** | **±51** | **1932** | **±66** | **1845** | **±81** |
| Water | 435 | ±41 | 440 | ±45 | 427 | ±83 | 454 | ±31 | 390 | ±36 | 547 | ±55 |
| *Bottled water* | 107 | ±20 | 92 | ±23 | 138 | ±39 | 138 | ±21 | 115 | ±24 | 172 | ±39 |
| *Tap water* | 328 | ±39 | 348 | ±44 | 289 | ±79 | 316 | ±28 | 275 | ±31 | 375 | ±50 |
| Milk & derivatives | 364 | ±20 | 348 | ±22 | 394 | ±39 | 222 | ±17 | 246 | ±25 | 187 | ±22 |
| Hot beverages | 141 | ±14 | 142 | ±17 | 137 | ±25 | 239 | ±19 | 209 | ±19 | 283 | ±37 |
| *Coffee* | 8 | ±4 | 12 | ±5 | 1 | ±1 | 59 | ±8 | 55 | ±10 | 66 | ±11 |
| *Tea* | 41 | ±7 | 43 | ±9 | 37 | ±13 | 61 | ±8 | 45 | ±9 | 85 | ±16 |
| *Mate* | 92 | ±12 | 87 | ±16 | 100 | ±20 | 119 | ±17 | 110 | ±16 | 132 | ±34 |
| *Other hot beverages* | 0 | ±0 | 0 | ±0 | 0 | ±0 | 0 | ±0 | 0 | ±0 | 0 | ±0 |
| SSB | 711 | ±46 | 748 | ±59 | 641 | ±73 | 818 | ±42 | 916 | ±56 | 674 | ±61 |
| *CSD* | 264 | ±22 | 266 | ±26 | 262 | ±43 | 411 | ±31 | 464 | ±43 | 333 | ±43 |
| *Juice-based drinks* | 352 | ±35 | 388 | ±46 | 282 | ±53 | 304 | ±27 | 329 | ±40 | 266 | ±31 |
| *Functional beverages* | 6 | ±2 | 5 | ±3 | 8 | ±5 | 16 | ±5 | 15 | ±6 | 17 | ±10 |
| *RTD tea & coffee* | 3 | ±1 | 1 | ±1 | 7 | ±4 | 1 | ±1 | 1 | ±1 | 1 | ±1 |
| *Flavored water* | 86 | ±18 | 88 | ±23 | 81 | ±32 | 86 | ±14 | 106 | ±21 | 58 | ±15 |
| 100% fruit juices | 19 | ±5 | 13 | ±4 | 32 | ±12 | 17 | ±5 | 18 | ±7 | 14 | ±5 |
| A/NSD | 133 | ±20 | 148 | ±28 | 105 | ±25 | 128 | ±19 | 126 | ±25 | 132 | ±28 |
| Alcoholic beverages | 1 | ±0 | 1 | ±1 | 1 | ±1 | 17 | ±5 | 26 | ±9 | 5 | ±3 |
| Other beverages | 2 | ±1 | 2 | ±1 | 1 | ±1 | 2 | ±1 | 2 | ±2 | 2 | ±2 |

*TFI* total fluid intake*, SSB* sugar sweetened beverages, *CSD* carbonated sweetened drinks, *RTD* ready to drink, *A/NSB* artificial/non-nutritive sweeteners beverages, *ND* no data

**Table S3d** Mean daily intake (SEM) of the different fluid types (mL/day) in Uruguay by sex and age category

|  | 6-9 years | | | | | | 10-17 years | | | | | |
| --- | --- | --- | --- | --- | --- | --- | --- | --- | --- | --- | --- | --- |
|  | Total | | Males | | Females | | Total | | Males | | Females | |
| **TFI** | **1693** | **±52** | **1804** | **±87** | **1594** | **±58** | **1668** | **±68** | **1745** | **±109** | **1587** | **±80** |
| Water | 535 | ±34 | 555 | ±50 | 518 | ±48 | 561 | ±51 | 586 | ±87 | 533 | ±53 |
| *Bottled water* | 439 | ±36 | 459 | ±50 | 421 | ±51 | 470 | ±51 | 518 | ±87 | 420 | ±51 |
| *Tap water* | 96 | ±20 | 96 | ±28 | 97 | ±29 | 90 | ±20 | 69 | ±23 | 113 | ±32 |
| Milk & derivatives | 418 | ±28 | 444 | ±48 | 395 | ±32 | 318 | ±26 | 327 | ±33 | 309 | ±42 |
| Hot beverages | 1 | ±1 | 0 | ±0 | 2 | ±2 | 164 | ±31 | 139 | ±40 | 190 | ±48 |
| *Coffee* | 0 | ±0 | 0 | ±0 | 0 | ±0 | 47 | ±12 | 38 | ±13 | 57 | ±20 |
| *Tea* | 0 | ±0 | 0 | ±0 | 0 | ±0 | 15 | ±6 | 7 | ±4 | 23 | ±11 |
| *Mate* | 1 | ±1 | 0 | ±0 | 2 | ±2 | 101 | ±27 | 93 | ±38 | 110 | ±39 |
| *Other hot beverages* | 0 | ±0 | 0 | ±0 | 0 | ±0 | 1 | ±1 | 1 | ±1 | 0 | ±0 |
| SSB | 687 | ±44 | 743 | ±74 | 638 | ±49 | 521 | ±41 | 611 | ±67 | 426 | ±43 |
| *CSD* | 382 | ±34 | 415 | ±58 | 352 | ±36 | 296 | ±30 | 344 | ±50 | 245 | ±33 |
| *Juice-based drinks* | 280 | ±34 | 286 | ±52 | 274 | ±44 | 186 | ±28 | 215 | ±43 | 156 | ±35 |
| *Functional beverages* | 0 | ±0 | 0 | ±0 | 0 | ±0 | 3 | ±1 | 4 | ±2 | 2 | ±1 |
| *RTD tea & coffee* | 0 | ±0 | 0 | ±0 | 0 | ±0 | 0 | ±0 | 0 | ±0 | 0 | ±0 |
| *Flavored water* | 26 | ±18 | 42 | ±38 | 12 | ±5 | 36 | ±9 | 48 | ±16 | 23 | ±10 |
| 100% fruit juices | 4 | ±4 | 8 | ±8 | 0 | ±0 | 21 | ±9 | 19 | ±12 | 23 | ±13 |
| A/NSD | 8 | ±5 | 11 | ±11 | 5 | ±3 | 57 | ±14 | 36 | ±12 | 79 | ±26 |
| Alcoholic beverages | 0 | ±0 | 0 | ±0 | 0 | ±0 | 2 | ±1 | 2 | ±2 | 1 | ±1 |
| Other beverages | 40 | ±13 | 44 | ±24 | 37 | ±11 | 25 | ±6 | 24 | ±7 | 26 | ±11 |

*TFI* total fluid intake*, SSB* sugar sweetened beverages, *CSD* carbonated sweetened drinks, *RTD* ready to drink, *A/NSB* artificial/non-nutritive sweeteners beverages, *ND* no data.

**Figure S1** Total fluid intake of children (4-9 years) and adolescents (10-17 years) expressed as a percentage (%) of the adequate intake of water from fluids set by EFSA, (6) based on 7-day mean of each participant by sex

*M* Males, *F* Females

**Figure S2a.** Distribution of daily total fluid intake (mL/day) among children (4-9years), by country

**Figure S2b.** Distribution of daily total fluid intake (mL/day) among adolescents (10-17years), by country

**Figure S3** Contribution of the different fluid types (%) to total fluid intake among children (4-9 years) and adolescents (10-17 years), by sex and country

*M* Males, *F* Females

**Figure S4** Percentage of children and adolescents drinking SSB on daily or less frequency, according to country and by sex

*M* Males, *F* Females
